# Combined effects of acidification and warming on soil denitrification and microbial community

**DOI:** 10.3389/fmicb.2025.1572497

**Published:** 2025-04-02

**Authors:** Peiyuan Xu, Mengke Gao, Yuchen Li, Jun Ye, Jianqiang Su, Hu Li

**Affiliations:** ^1^State Key Laboratory for Ecological Security of Regions and Cities, Institute of Urban Environment, Chinese Academy of Sciences, Xiamen, China; ^2^School of Life Sciences, Hebei University, Baoding, China; ^3^College of Juncao Science and Ecology, Fujian Agriculture and Forestry University, Fuzhou, China; ^4^University of Chinese Academy of Sciences, Beijing, China

**Keywords:** acidification, warming, RNA level, microbial community, denitrification rate

## Abstract

In light of the challenges posed by contemporary global warming and soil acidification, the respective effects of pH and temperature on soil microbiome and functions have been explored. However, the combined influence of acidification and warming on soil denitrification and active microbial communities are still unclear. Here, we conducted a microcosm experiment to investigate the influences of increasing temperature and acidification on active microbes such as bacteria and eukaryotic microbes. Denitrification rate in soil were detected using a C_2_H_2_ inhibition method. The results showed that the Shannon index of bacterial communities exhibited significant enhancement in response to warming and acidification, whereas their community patterns were predominantly shaped by pH. For the micro-eukaryotic community, temperature emerged as the main driver of variations in the α-diversity, with the MT group exhibiting significantly lower Shannon indices compared to LT and HT groups. Both pH and temperature exerted a combined effect on their community patterns. Additionally, pH was detected as a crucial factor influencing denitrification rates, with a significant negative correlation between pH and denitrification rate within the pH range of 4.32–7.46 across all temperatures in this study. Our findings highlighted the significant impacts of acidification on soil denitrification rates and active microbes under global warming, which provided an important scientific basis for agricultural production management and environmental protection in the context of global climate warming.

## 1 Introduction

Nitrous oxide (N_2_O) is a major greenhouse gas and is regarded as the most significant contributor to ozone depletion in the 21st century (Ravishankara et al., 2009; Montzka et al., 2011). As an essential component of the global biogeochemical cycle, soil serves as both the source and sink of atmospheric N_2_O (Ryden, 1981). Consequently, N_2_O released by soils, especially farmland soils, has received much attention in previous studies (Bhattarai et al., 2021), which have shown that microbial processes are critical to nitrous acid emissions from agricultural soils.

Denitrification is an important microbial process converting nitrate (NO_3_^–^) and nitrite (NO_2_^–^) into N_2_O and N_2_ in different ecosystems, for example, soil, sediment, and water (Long et al., 2013; Margalef-Marti et al., 2024). This process has been extensively studied in bacteria, showing as multiple reduction steps catalyzed by various enzymes, including nitrate reductase (NAR), nitrite reductase (NIR), nitric oxide reductase (NOR), and N_2_O reductase (NOS) (Sennett et al., 2024). In addition to bacteria, eukaryotic microbes represent a significant component of the soil microbial community, playing vital ecological roles in nitrogen processes (Mothapo et al., 2013, 2015; Maeda et al., 2015; Higgins et al., 2016). Differed from bacteria, several denitrifying eukaryotic microbes, such as *Fusarium oxysporum, Bolivina plicata*, and *Stainforthia* sp. (Kobayashi et al., 1996; Kamp et al., 2015), lack the *nosZ* gene, resulting in N2O as the final denitrification product (Laughlin and Stevens, 2002; Crenshaw et al., 2008). Soil environments would affect the relative contributions of bacteria and eukaryotic microbes to denitrification, especially N_2_O emission rates in soil. Eukaryotic microbes derived N_2_O emissions are comparable to, or even exceeded, those from bacterial denitrification (Chen et al., 2014).

Complex relationships between soil pH and denitrification processes have been determined in a previous study (Firestone et al., 1980). The ratios of denitrification products was strongly influenced by pH (ŠImek and Cooper, 2002), specifically, the N_2_O/(N_2_O + N_2_) ratio seemed to have a negative correlation with soil pH in agricultural settings pH 5–8 (Bakken et al., 2012). Additionally, there was an increase in the N_2_O/(N_2_O + N_2_) ratio during denitrification under acidic conditions (pH < 5.0). Raising soil pH to near-neutral levels (pH > 6.5) through liming can reduce N_2_O emissions; However, increasing the pH of acidic soils (pH < 5.6) to moderately acidic levels (pH 5.6–6.0) generally led to higher N_2_O emissions. A hump-shaped relationship existed between soil pH and N_2_O, leading to peak N_2_O emissions at moderate soil acidity (Qiu et al., 2024). Furthermore, variations in pH over both short and long terms affect soil N_2_O emissions differently, as the dominant microbial communities can shift due to pH-induced changes in the microbial source of N_2_O (Baggs et al., 2010). Additionally, temperature has also been indicated to be important factor affecting the distribution of soil denitrifying bacterial communities (Braker et al., 2010; Taylor et al., 2019). A previous study has demonstrated that ammonia oxidizers and bacterial denitrifiers were significantly inhibited at high temperatures, whereas micro-eukaryotic denitrifiers are well-adapted and may be the primary contributors to N_2_O emissions in acidic soils (Xu et al., 2017). Whereas, most of previous studies investigating pH or temperature impacts on microbes determined the community and abundance of microbes at DNA level, but not RNA level, which reflects active microbes in different ecosystems. Previous studies have determined the correlation between denitrification rate and microbial community including microbial abundance and diversity at DNA level (Yao et al., 2013; Wang et al., 2014; Chunyi et al., 2024). However, active microbes are the drivers of nutrient transformations in different ecosystems. The effects of pH and temperature on active microbial community are still limited, especially the combined impacts of pH and temperature.

To explore the combined effects of warming and acidification on soil denitrification rates and the active microbial communities, we conducted a microcosm experiment with a gradient of pH (4.9–7.7) and soil temperature (20°C–30°C). Additionally, we employed transcriptomic methods to analyze RNA levels, allowing us to investigate the relationship between denitrification rates and the dynamics of active microbial communities. This study will shed light on how active microorganisms, alongside soil nutrients, influence soil denitrification, offering insights into the combined effects of soil acidification and rising temperature on greenhouse gas production.

## 2 Materials and methods

### 2.1 Soil microcosm designment

Soil samples with an initial pH of 4.95 were collected from a tea garden in Ningbo, China (121.86°E, 29.75°N). Soil was homogenized by sieving through a 2 mm mesh. The soil samples were maintained at room temperature with a 20% moisture content for 30 days to stabilize the soil properties and microbial community. Limestone (CaCO_3_, 99.0%, AR) was added to adjust the soil pH values. During the soil pH adjustment period, three pH levels were established as high pH 7.7 (HP), medium pH 6.4 (MP) and low pH 4.9 (LP), respectively. The soil with stabilized pH were incubated at three different soil temperature gradients (soil temperature was measured by using a thermometer) for 30 days, including low temperature 20°C (LT), medium temperature 25°C (MT), and high temperature 30°C (HT), respectively. During the adjustment of pH and temperature, the moisture content was maintained at 20%, consistent with level during previous stable incubation. Finally, a total of nine experimental treatments were prepared: LTHP, MTHP, HTHP, LTMP, MTMP, HTMP, LTLP, MTLP, and HTLP. Each treatment consisted of three replicates, with each replicate containing 200 g of soil. pH decreased slightly after 1 month of incubation (Supplementary Table 1).

### 2.2 Analysis of soil physicochemical property

The concentration of ammonia (NH_4_^+^) and nitrate (NO_3_^–^) were determined using a continuous flow analyzer (AA3 analyzer, German) after extracted by 1 mol/L KCl solution (soil:KCl solution = 1:10) (Li et al., 2025). The pH of each soil was measured using an XL60 pH meter (Fisher Scientific, United States) after being suspended in deionized water (ddH_2_O) with 1:2.5 soil-to-water ration (Li et al., 2016). Soil moisture was calculated after dried in an oven at 105°C for 16 h. Total carbon, nitrogen, and sulfur contents were analyzed using a Vario MAX CNS elemental analyzer (ELEMENTAR, German) (Xu et al., 2014).

### 2.3 Determination of denitrification rate

Denitrification rate was measured using an acetylene (C_2_H_2_) inhibition method in accordance with a previous study (Xu et al., 2019). In brief, 10 g of fresh soil was placed in a 120 mL serum bottle with 5 mL 2.4 mM NaNO_3_ and 5 mL 0.06 M glucose. The serum bottles were sealed with rubber stoppers and were alternately vacuumed and flushed with helium (He) gas to establish anaerobic conditions. For determination of potential N_2_O production rate, 10% (v/v) of C_2_H_2_ was added to inhibit the reduction of N_2_O to N_2_. The rates of N_2_O production in the treatments without C_2_H_2_ were calculated as the real denitrification rates (Philippot et al., 2011). The concentration of N_2_O in the headspace was measured at 1 and 5 h (Supplementary Table 2) using a gas chromatograph (7890A; Agilent Technologies, Santa Clara, CA, United States) (Molstad et al., 2007; Xu et al., 2019).

### 2.4 RNA extraction, reverse transcription, and target-gene sequencing

Total RNA was extracted from 2 g fresh soil using a RNeasy PowerSoil Total RNA Kit (Qiagen) according to the manufacturer’s instructions, and the purified RNA without DNA was stored at −*80*°C until used. Complementary DNA (cDNA) was synthesized through reverse transcription using an ABKscript RT MasterMix (OneStep gDNA Removal) Kit, and the resulting cDNA was used as template for target gene amplification. To evaluate the communities of bacteria and eukaryotic microbes in different treatments, we performed PCR amplification for bacterial 16S rRNA gene and micro-eukaryotic 18S rRNA gene using primer set of 338F/806R (Yang et al., 2020) and 565F/981R (Salmaso et al., 2020), respectively. The amplicons were purified using a E.Z.N.A.^®^ Gel Extraction Kit (Omega, United States) and sent to Magigene Biotechnology Co. (GuangZhou, China) for high-throughput sequencing on a Novaseq 6000 PE250 platform.

### 2.5 Data processing and statistical analysis

We utilized Quantitative Insights Into Microbial Ecology version 2 (QIIME2) (Bolyen et al., 2019) to analyze the sequences, and employed the DADA2 plugin (Callahan et al., 2016) to denoise sequences and generate Amplicon Sequence Variants (ASV) approximately 250 base pairs in length. For both bacteria and eukaryotic microbes, ASVs with only one sequence were discarded in the following analysis (Li et al., 2023). SILVA 138 SSU Ref NR99 and RDP 18S v4.1 database (Pruesse et al., 2007; Wang et al., 2007) were used for the classification of the bacterial and micro-eukaryotic taxonomy, respectively. ASVs identified as mitochondria and chloroplast sequences were removed. Alpha diversity indices (Shannon and Chao1) were calculated based on species richness to assess the biodiversity of microbial communities (Supplementary Table 3). Principal Co-ordinates Analysis (PCoA) were constructed to exhibit the distribution patterns of microbial (i.e., bacteria and eukaryotic microbes), bacterial, and micro-eukaryotic communities based on Bray-Curtis distances. Then, redundancy analysis (RDA) was selected to distinguish the soil properties affecting microbial communities. The relative abundances of bacterial and micro-eukaryotic species were displayed using heatmap plot using R with “pheatmap” package (version 1.0.12). We used LDA Effect Size (LEfSe) to identify species with significant differences between treatment via the website^1^, with an LDA threshold of four and a *p*-value threshold of 0.05. Additionally, classes differences between bacterial and micro-eukaryotic active microorganisms under different treatments were assessed using two-way ANOVA to extract F values for evaluating the impact magnitude (Package “vegan” v2.6–6.1). After filtering for ASVs with relative abundance greater than 0.01%, a heatmap was generated using the top 30 species by relative abundance at the class level. Mantel test and Pearson’s correlation analyses were conducted using R with “LinkET” package (version 0.0.7.4). The *p*-values of mantel test were adjusted using FDR. The partial least squares path modeling (PLSPM) was constructed to explore the mechanism of effects of microbial alpha and community pattern, total nutrient (including TS, TC, and TN), inorganic nitrogen (including NO_*x*_-N, and NH_4_^+^-N), pH, and temperature on soil denitrification rates using the “plspm” package (version 0.5.1) in R software. Multiple goodness of fit criteria was tested for the model as follows: Goodness of Fit (GoF > 0.6), Dillon-Goldstein’s rho (DG.rho > 0.7), Average Variance Extracted (AVE > 0.5). Random forest analyses were conducted using the “rfPermute” package (version 2.5.2) in R with 1,000 permutations and 500 decision trees. The data for Random Forest analysis incorporated actual denitrification rates (measured without C_2_H_2_ inhibition), soil physicochemical parameters, microbial diversity and community patterns (PCoA 1 axis). All data used R software (R4.3.1) for statistical testing and correlation analysis. Tukey-HSD was used for *post hoc* pairwise comparisons. The date for bacterial 16S rRNA genes and micro-eukaryotic 18S rRNA genes could be downloaded from ScienceDB using https://doi.org/10.57760/sciencedb.17932.

## Results

### 3.1 Denitrification rates

**FIGURE 1:**
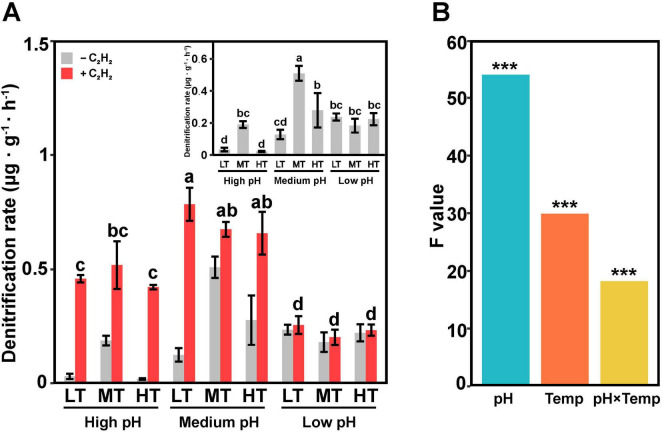
N_2_O emission from soil samples with different treatments. **(A)** N_2_O emission rates under different conditions: with (red bars) and without (gray bars) C_2_H_2_. **(B)** Factors, i.e., pH and Temp (temperature), influencing denitrification rates. The treatment groups g low temperature (LT), medium temperature (MT) and high temperature (HT) in the figure represented low temperature, medium temperature and high temperature, respectively. These letters employ the alphabet mark method to indicate statistically significant differences in multiple comparisons. The symbol ‘***’ represents asterisks for significance in figures.

Significant differences in denitrification rates were observed among the various treatment groups, with rates ranging from 0.018 to 0.55 μg⋅g^–1^⋅h^–1^ in the absence of acetylene (C_2_H_2_) and N_2_O production rates ranging from 0.17 to 0.87 μg⋅g^–1^⋅h^–1^ in treatments with C_2_H_2_ (Figure 1A). In the treatment with C_2_H_2_, denitrification rates were significantly higher (*p* < 0.05) in the medium temperature and medium pH (MTMP) treatment than in other treatments. Conversely, high pH significantly decreased the denitrification rates in soils, especially treated with low and high temperature (*p* < 0.05). In the treatment without C_2_H_2_, the highest N_2_O production rate was observed in the LTMP treatment, while the lowest rate was found in the MTLP treatment. Additionally, N_2_O production rates in middle pH groups were the highest, followed by in high pH and low pH (*p* < 0.05). Calculating based on N_2_O emission in treatments with and without C_2_H_2_, the rates of N_2_O reduction, i.e., conversion of N_2_O to N_2_, were the lowest in low pH treatments compared in high and middle treatment groups (Supplementary Table 2).

Soil pH, temperature, and combination of pH and temperature significantly (two-way ANOVA test, *p* < 0.001) affected denitrification rates in soil. Notably, soil pH played a more important role in regulating soil denitrification rate in comparison with temperature (Figure 1B).

### 3.2 Diversity of active microbial communities

A total of 50,610 bacterial ASVs and 6,181 micro-eukaryotic ASVs were obtained in soil samples based on high-throughput sequencing of bacterial 16S rRNA genes and micro-eukaryotic 18S rRNA genes at RNA-level. The coverage of 99.20% and 99.64%, for bacterial and micro-eukaryotic communities, respectively, indicated sufficient sampling depth to capture overall microbial diversity across all 27 samples. Significant acidic and thermal variations (*p* <0.05) were observed in the Shannon index (Supplementary Figures 1A, C, E), with HT and LP treatments exhibiting higher values than LT and HP treatments (Supplementary Figure 1), indicating that pH and temperature changes played a pivotal role in shaping the alpha diversity of bacteria. While temperature mainly affected the alpha diversity of soil eukaryotic microbes (Supplementary Figures 2A, E), with HT and LT treatment showing higher values than MT treatment (Supplementary Figure 2). From the perspective of overall active microorganisms, temperature primarily had a significant effect on alpha diversity (Supplementary Figures 3A–E).

**FIGURE 2:**
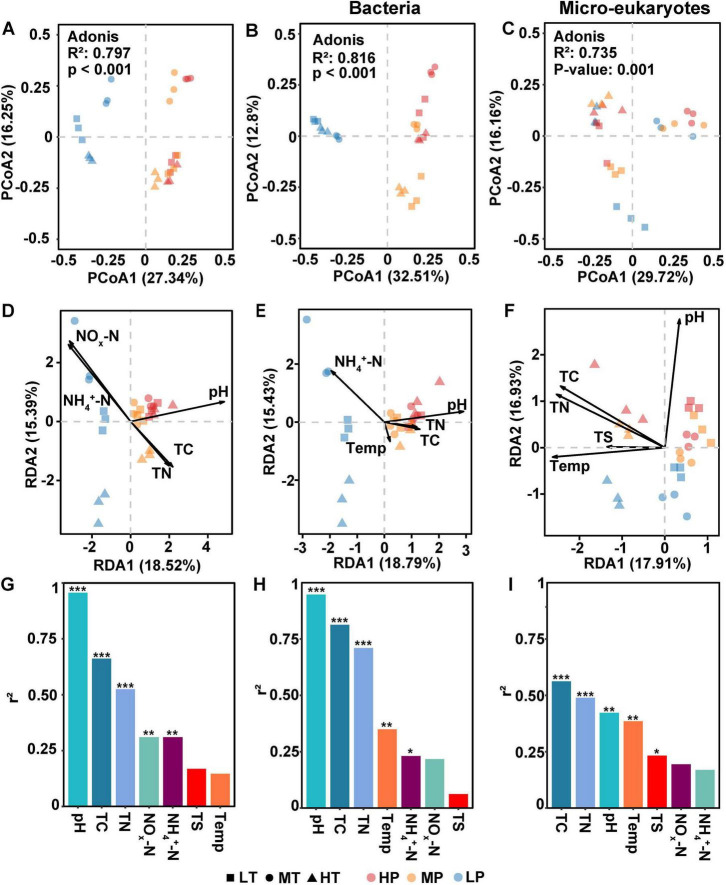
Distribution patterns of microbial communities and factors influencing microbial communities in soil samples. **(A–C)** Principal Co-ordinates Analysis (PCoA) of microbial communities, bacterial and micro-eukaryotic communities, respectively. **(D–F)** Environmental factors affecting microbial communities, bacterial communities, and micro-eukaryotic communities, respectively. **(G–I)** Correlation coefficient (r^2^) of environmental factors to microbial communities, bacterial communities, micro-eukaryotic communities, respectively, calculated by redundancy analysis (RDA) analysis. The environmental factors included pH, temperature (Temp), total carbon (TC), total nitrogen (TN), total sulfur (TS), nitrate and nitrite nitrogen (NO_*x*_-N), and ammonium nitrogen (NH_4_^+^-N). The symbol ‘*’, ‘**’, ‘***’ represents asterisks for significance in figures.

PCoA plots showed that significant differences were observed in community patterns of microbes including bacteria and eukaryotic microbes (Figure 2A) among various treatments (adonis R^2^ = 0.80, *p* < 0.001). Further, we found that microbial profiles in low pH (LP) treatments differed from those in middle (MP) and high (HP) groups. Moreover, pH changed the distribution patterns of microbes in soil samples across different pH (Figure 2A). Similarly, bacterial patterns in soil samples treated with low pH were separated from both MP and HP treatments at axis one, which explained 32.51% variation in bacterial communities (Figure 2B). In contrast, the micro-eukaryotic patterns were separated at axis 1 based on temperature, and three groups of micro-eukaryotic communities in soils with different temperatures were clearly separated (Figure 2C).

RDA analysis determined the factors influencing microbial communities (Figure 2D), bacterial communities (Figure 2E), and micro-eukaryotic communities (Figure 2F) in soil samples, respectively. pH, TC, TN, NO_*x*_-N, and NH_4_^+^-N were determined to significantly affect microbial communities (*p* < 0.01). Further, we found that pH played the most important role on regulating microbial community patterns in soil samples (Figure 2G). For bacterial community, pH, TC, TN, temperature, and NH_4_^+^-N concentration were detected as environmental factors significantly influencing their community structures (*p* <0.05). Correlation coefficient analysis further indicated that pH acted as the most important factor in changing bacterial communities, followed by TC, TN, and temperature (Figure 2H). Finally, TC, TN, TS, pH and temperature significantly influenced micro-eukaryotic communities, with pH and temperature showing equal contributions (Figure 2I).

### 3.3 Variant microbial species among treatments

**FIGURE 3:**
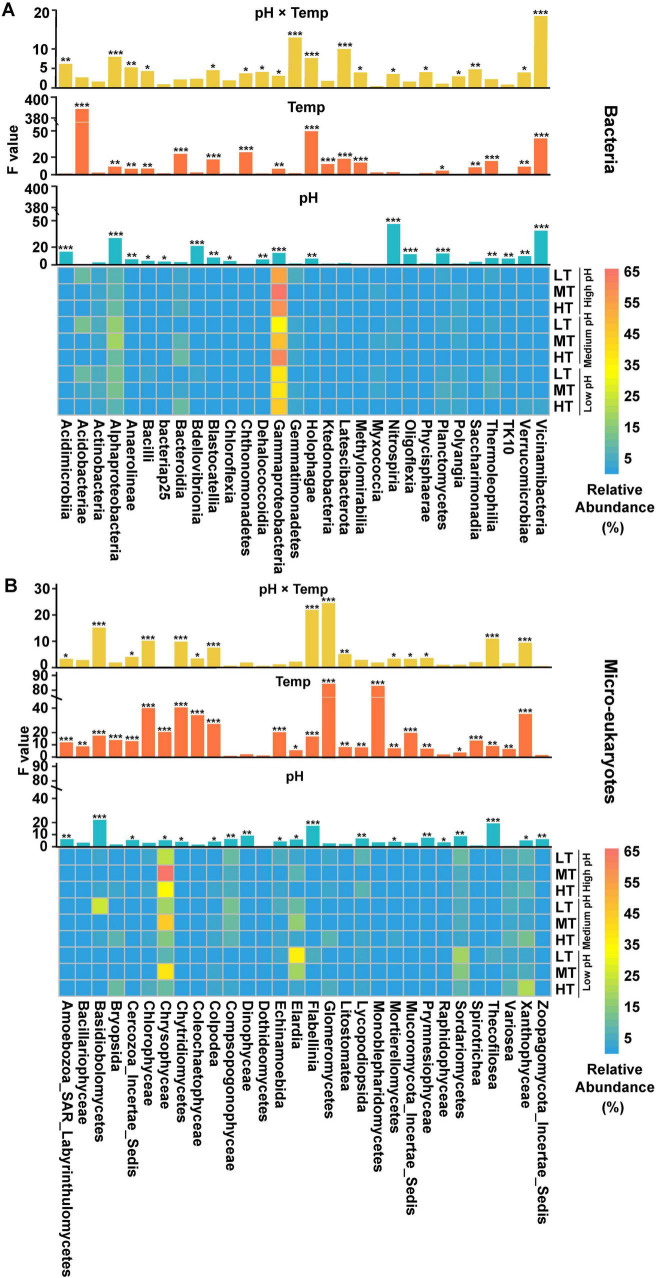
Factors influenced the relative abundance of variant taxa among different treatments, including **(A)** active bacterial microorganisms and **(B)** eukaryotic active microorganisms. The symbol ‘*’, ‘**’, ‘***’ represents asterisks for significance in figures.

The genus-level classification plots were shown in Supplementary Figures 4A, 5A. LEfSe analysis revealed that 62 bacterial and 77 micro-eukaryotic species exhibited significant differences (Supplementary Figures 4B, C, 5B, C) among treatments (*p* < 0.05, standardized scaling factor: 1000000). Among the top 30 bacterial classes, most showed significant differences (Figure 3A), with 18 classes significantly affected by both pH and temperature, including Alphaproteobacteria, Holophagae, and Vicinamibacteria. The acidification process enriched several bacteria, such as Acidimicrobiia (HP: 0.49%, MP: 0.51%, LP: 0.90%), Dehalococcoidia (HP: 0.072%, MP: 0.084%, LP: 0.15%) and Bdellovibrionia (HP: 0.37%, MP: 0.55%, LP: 2.56%). In addition, the warming process enriched several bacteria, such as Acidobacteriae (HP: 4.60%, MP: 4.77%, LP: 5.01%) and Bacteroidia (LT: 1.76%, MT: 5.11%, HT: 8.29%).

Among the top 30 micro-eukaryotic classes, results showed temperature played a more important role in regulating the abundance of eukaryotic microbes in comparison with pH (Figure 3B). There were 25 classes of eukaryotic microbes significantly affected by temperature, such as Glomeromycetes (LT: 2.25%, MT: 1.35%, HT: 5.98%), Monoblepharidomycetes (LT: 0.22%, MT: 0.11%, HT: 0.73%) and Coleochaetophyceae (LT: 0.71%, MT: 0.21%, HT: 1.16%). In addition, Elardia reached its peak under LT groups (LT: 16.28%, MT: 14.18%, HT: 2.68%) and Chrysophyceae exhibited the highest relative abundance under MT treatment (LT: 16.18%, MT: 50.11%, HT: 19.88%).

### 3.4 Factors affecting denitrification and N_2_O production rates

**FIGURE 4:**
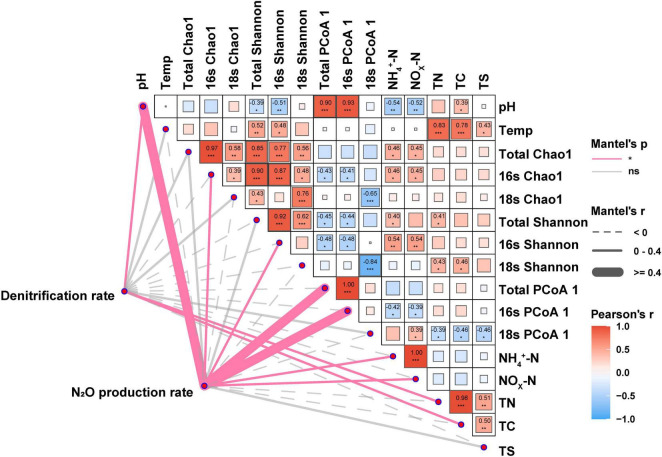
Mantel test analysis identified factors influencing both denitrification and N_2_O production rate. The Mantel’s r and *p*-values indicated the correlation and significance, respectively. Dashed lines and solid lines indicated negative and positive correlations, respectively, while red and gray color meant significant (*p* < 0.05) and non-significant, respectively. Total Chao1: Chao1 index of microbial including bacterial and micro-eukaryotic community. 16S Chao1 and 18S Chao1: Chao1 index of bacterial and micro-eukaryotic community, respectively. Total Shannon: Shannon index of microbial including bacterial and micro-eukaryotic community. 16S Shannon and 18S Shannon: Shannon index of bacterial and micro-eukaryotic communities, respectively. Total β diversity: PCoA1 axis index of microbial including bacterial and micro-eukaryotic community. 16S β diversity and 18S β diversity were used to represent bacterial and micro-eukaryotic PCoA1 axis, respectively. The symbol ‘*’, ‘**’, ‘***’ represents asterisks for significance in figures.

Mantel tests were employed to detect the biotic and abiotic factors affecting denitrification and N_2_O production rates in soil and to analyze the relationships between biotic and abiotic factors (Figure 4). The results showed that pH (r = 0.68, *p* < 0.05), active bacterial diversity (Chao1 and Shannon: r = 0.22, *p* < 0.05 and r = 0.18, *p* < 0.05, respectively), community pattern (microbes and bacteria: r = 0.71, *p* < 0.05 and r = 0.74, *p* < 0.05, respectively), inorganic nitrogen (NH_4_^+^ and NO_x_^–^: r = 0.33, *p* < 0.05 and r = 0.32, *p* < 0.05, respectively) significantly influenced the rates of N_2_O production. Differing from N_2_O production, denitrification rate was significantly affected by pH (r = 0.26, *p* < 0.05), total carbon (TC, r = 0.25, *p* < 0.05), and total nitrogen (TN, r = 0.29, *p* < 0.05). Notably, no significant correlation between temperature and denitrification rate, and between temperature and N_2_O production rate was observed, while the significant correlations between temperature and microbial alpha diversity, and soil nutrients (e.g., TC. TN, and TS) were observed in this study.

**FIGURE 5:**
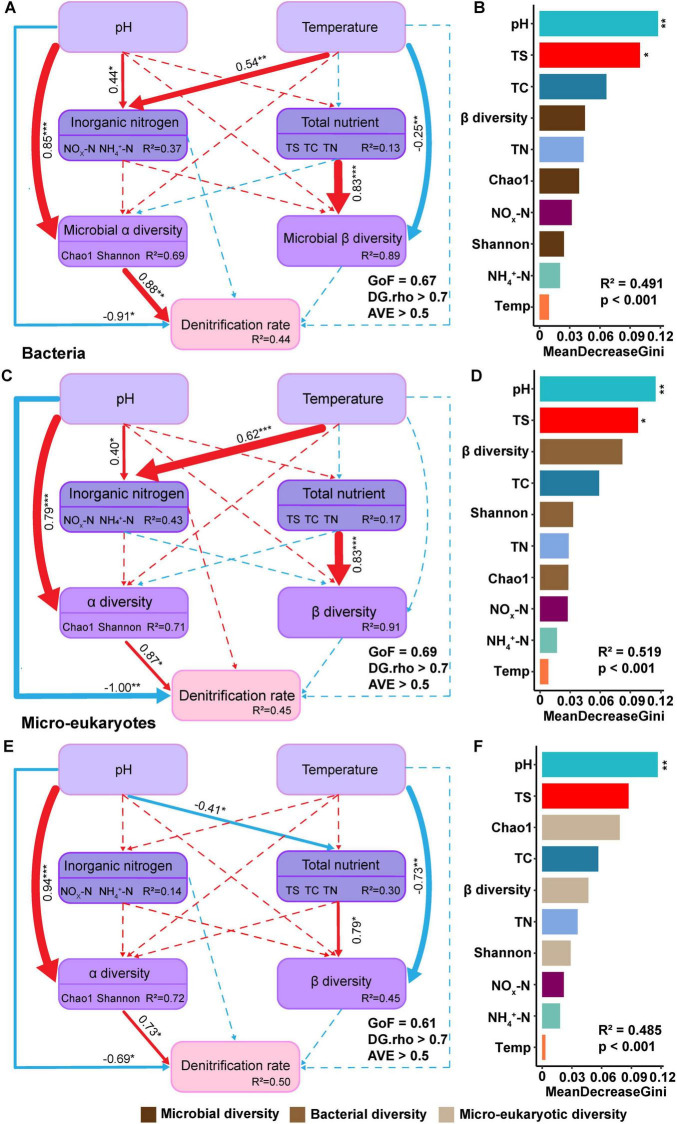
Partial least squares path modeling (PLSPM) model elucidated the influence of microbial and soil properties on denitrification rate. The model assessed the impacts of active soil microorganisms (bacteria and eukaryotic microbes) and physicochemical properties on denitrification rate. **(A,C,E)** The fitness of PLSPM model was acceptable (GoF > 0.6, DG.rho > 0.7, AVE > 0.5). Microbial alpha and beta diversity represented the overall diversity of bacterial and eukaryotic active microbes. Positive correlations were depicted by red solid lines, and negative correlations by blue solid lines. Dashed lines indicated non-significant correlations, with the color corresponding to the correlation status. Path coefficients and coefficients of determination (R^2^) were calculated after 999 bootstrapping iterations, and all path coefficients shown were statistically significant (*p* < 0.05). **(B,D,F)** Random Forest modeling with permutation tests (*n* = 999, *p < 0.05, **p < 0.01) indicated that the MeanDecreaseGini (MDG) values showed the significant importance of environmental variables and microbial biodiversity indices. **(A,B)** Represented overall microorganisms. The index of β diversity, Chao1 and Shannon indicated microbial communities including bacterial and micro-eukaryotic community diversity. **(C,D)** Denoted bacteria. Similarly, the index of β diversity, Chao1 and Shannon indicated bacterial community diversity. **(E,F)** Referred to eukaryotic microbes and their indexes of β diversity, Chao1 and Shannon indicated micro-eukaryotic community diversity. The environmental factors included pH, temperature (Temp), total carbon (TC), total nitrogen (TN), total sulfur (TS), nitrate and nitrite nitrogen (NO_*x*_-N), and ammonium nitrogen (NH_4_^+^-N). The symbol ‘***’ represents asterisks for significance in figures.

PLSPM was employed to further analyze the mechanisms of the effects of pH and temperature on soil denitrification rate and model explained 44%, 45%, and 50% of the variation in denitrification rate for overall active microbial, bacterial and micro-eukaryotic groups, respectively (Figures 5A, C, E). The alpha diversity of active microorganisms exerts the strongest positive effect on denitrification rates. Furthermore, inorganic nitrogen has the opposite effect on denitrification rates in both bacterial and micro-eukaryotic models. Notably, pH has a greater influence on denitrification rates than temperature (Supplementary Figures 6A–C). Consistent with the Mantel test results, pH exerted a stronger influence on denitrification rates than temperature (Figures 5B, D, F). Alpha diversity had the pH directly and indirectly affected the rates of denitrification through altering soil properties, bacterial alpha diversity (Figure 5C), and micro-eukaryotic community pattern (Figure 5E). Temperature, conversely, only indirectly affected denitrification rates by influencing inorganic nitrogen (NH_4_^+^ and NO_*x*_^–^) concentrations and microbial community pattern (Figures 5A, C, E). Additionally, increasing temperature significantly and directly affected the community compositions of eukaryotic microbes but not bacteria in soil ecosystems (Figures 5C, E). Further, a Random Forest analysis corroborated these findings, indicating that pH was the most significant factor influencing denitrification rates in both micro-eukaryotic and bacterial groups, while the importance of other physicochemical properties, including TC and TN, ranked secondary in comparison, consistent with the Mantel test results (Figures 5B, D, F).

## 4 Discussion

### 4.1 The impacts of bacteria on N_2_O emission in soil

In the present study, we observed that the relative abundance of denitrifying bacteria, such as *Pseudoxanthomonas*, *Bacillus*, and *Pseudomonas* (Hartmann and Six, 2023), exhibited relatively high abundances under MTMP treatment, and this treatment had the highest denitrification rate. Denitrifying bacteria are abundant in various soil types, including farmland, parks, and tea gardens, where they enhance N_2_O emissions and play a crucial role in the nitrogen cycle (Crenshaw et al., 2008; Hiis et al., 2024; Tan et al., 2024). Additionally, there was a significant positive correlation between inorganic nitrogen levels and bacterial diversity (Figure 4), which may be attributed to the nitrification processes performed by nitrifying bacteria. Nitrifying bacteria, such as *Nitrosospira* and *Nitrospira* (Zhang et al., 2022; Deng et al., 2024), exhibited the highest abundance under MTMP treatment. This condition also led to the highest denitrification rates by providing substrates for denitrification and enhancing the activity of associated microorganisms (Su et al., 2021). These findings highlight the significant role of active bacteria in mediating biochemical processes and influencing soil multifunctionality.

### 4.2 Potential impacts of micro-eukaryotic denitrifiers on N_2_O emission in soil

Micro-eukaryotic denitrifiers play an important role in N_2_O emission (Figures 4, 5E). Although the richness of micro-eukaryotic denitrifiers was relatively rare compared to their bacterial counterparts (Bösch et al., 2023), they exhibited biome-specific differences in both relative abundance and species distributions (Figure 2C and Supplementary Figure 5A). Studies indicate that fungi contribute more to soil N_2_O emissions than bacteria in acidic soils (Laughlin and Stevens, 2002; Herold et al., 2012; Zheng et al., 2020; Xiong et al., 2024), especially those belonging to the genus *Fusarium*, can perform denitrification *sensu stricto* (Keuschnig et al., 2020), resulting in an increase in N_2_O emissions (Shoun et al., 1989; Zheng et al., 2020). As studies have shown that *Fusarium* is an important fungus in N_2_O emission (Maeda et al., 2015; Zheng et al., 2020; Shao et al., 2024), under acidic conditions (e.g., in LTLP treatment), the relative abundance of *Fusarium* is highest, which may explain the phenomenon that soil still retains denitrification capacity (Figure 1A) even in strongly acidic environments (pH 4.3–4.7) (Chen et al., 2014). In addition to fungi, other eukaryotic microorganisms, mainly including foraminifers such as *Globobulimina pseudospinescens*, *Bolivina plicata* and *Stainforthia sp.* (Kamp et al., 2015; Risgaard-Petersen et al., 2006), are also involved in denitrification processes that release N_2_O. Unlike active bacteria, eukaryotic microbes exhibit similar responses to pH and temperature (Figures 2I, 5E), demonstrating their ability to thrive under extreme climatic conditions with high temperatures (Xu et al., 2017). The relatively low sensitivity of micro-eukaryotic richness to pH and temperature changes compared to bacteria may explain their stability in denitrification processes (Huang et al., 2017; Banerjee et al., 2024). These results imply potential micro-eukaryotic contributions to nitrogen cycling processes, laying the foundation for future use of eukaryotic microbes to improve soil health.

### 4.3 The potentially greater role of pH than temperature in changing N_2_O emission and active microorganism communities in soil

Temperature is a critical factor influencing denitrification processes (Barnard et al., 2005). Elevated temperatures (typically 20°C–25°C) enhance N_2_O emissions (Dai et al., 2020). This phenomenon can be attributed to multiple mechanisms. Higher temperatures accelerate the mineralization process (Dai et al., 2020), which elevates soil inorganic nitrogen levels (Figure 5A) and supplies additional substrates for microbial nitrification and denitrification, thereby promoting N_2_O production. Furthermore, rising temperatures stimulate the activity and growth of denitrifying microorganisms in soil (Schulz et al., 2017). For instance, warmer conditions promote the proliferation of *Bacillus* (Choma et al., 2000), directly contributing to increased N_2_O emission.

This study reveals a negative correlation between soil pH and denitrification rate, aligning with earlier report (Čuhel and Šimek, 2011). Previous research demonstrated that pH dominates the ratio of denitrification products (Figures 2G–I, 4, 5B, D, F), with N_2_O being the primary product at pH 4.6–5.4 (Koskinen and Keeney, 1982). This phenomenon may be attributed to the significantly negative correlation between pH and inorganic nitrogen concentrations (Figure 4). Specifically, soil acidity enhances the effect of nitrate on the composition denitrification-derived gaseous products, driving higher N_2_O production at pH 4.9 than at pH 6.5 (Firestone et al., 1980). However, reduced pH hinders the decomposition of soil organic nitrogen (Li et al., 2018), partially suppressing denitrification rates. pH further indirectly inhibits denitrification (Krichels et al., 2025) by altering active microbial communities (Figures 2G–I). The limited N_2_O reduction in LP groups (Figure 1A) implies that acidic conditions may inhibit bacterial N_2_O reductase activity, potentially due to the sensitivity of reductase translation and assembly to pH value (ŠImek and Cooper, 2002). Active bacteria responses to pH fluctuations are rapid, as their lack of a cytoskeleton contrasts with the structural rigidity conferred by fungal chitinous cell walls (Wang and Kuzyakov, 2024). Additionally, fungal spore formation further reinforces resistance to stressors such as drought and acidification (Yang et al., 2024).

Soil pH exerts a stronger impact than temperature on N_2_O emission and active microorganisms (Figures 2–5), particularly in shaping the denitrifying bacterial community patterns (Lauber et al., 2009; Bakken et al., 2012). This likely arises because pH more robustly governs nitrate utilization by denitrifiers (Blackmer and Bremner, 1978; Senbayram et al., 2019). Our analysis demonstrated a significant negative correlation between nitrate and pH, but no such relationship with temperature (Figure 4). Elevated nitrate levels, as a substrate, promote the proliferation of *nirK-* and *nirS-* type denitrifiers (Hao et al., 2022). Moreover, pH exerts a marked effect on microbial diversity (Figures 4, 5) and community patterns (Figure 2), particularly on certain bacterial denitrifiers (Pan et al., 2023). Researchers revealed that significant changes in the proteome of denitrifier (e.g., *P. denitrificans*) were identified when comparing pH 6.5 and 7.2, exhibiting significant downregulation of functional proteins (Olaya-Abril et al., 2021). These findings suggest that pH stability, compared to temperature, exerts a stronger influence on the survival of denitrifiers, ultimately shaping microbial diversity and community patterns. This may account for the reduced relative abundance of *Pseudomonas* and *Pseudoxanthomonas* at low pH, as observed in genus-level bacterial community structure analyses.

### 4.4 Research limitations

While this study demonstrated that the effects of soil acidification and warming on denitrification processes through RNA-seq approaches, it should be noted that the current conclusions were exclusively based on correlation analyses, such as RDA, Mantel test and PLSPM model. To clarify the mechanisms on how active microorganisms impacts soil nitrogen cycling processes, future studies should consider combining quantitative PCR and metatranscriptomics to systematically assess the functional contributions of microbial communities (e.g., *nirK*, *nirS*, and *nosZ* genes) in soil denitrification processes.

## 5 Conclusion

In summary, the results of this study demonstrated a stronger impact of pH on denitrification rates than temperature. Micro-eukaryotic and bacterial communities exhibited distinct responses to soil acidification and warming. Bacterial communities were predominantly shaped by pH, while micro-eukaryotic communities were influenced similarly by pH and temperature. Additionally, we found that micro-eukaryotic active microbes also contributed to the denitrification and N_2_O emission in the used soil of this study. These findings highlighted the important role of pH on nitrogen cycling in soil through changing active bacterial and micro-eukaryotic communities and provided scientific basis for agricultural management during the global warming.

## Data Availability

The datasets presented in this study can be found in online repositories. The names of the repository/repositories and accession number(s) can be found here: https://www.scidb.cn/en, https://doi.org/10.57760/sciencedb.17932.

## Footnotes

1
https://www.bic.ac.cn/BIC

## References

[B1] Baggs E. M. Smales C. L. Bateman E. J. (2010). Changing pH shifts the microbial sourceas well as the magnitude of N_2_O emission from soil. *Biol. Fertil. Soils* 46 793–805. 10.1007/s00374-010-0484-6

[B2] Bakken L. R. Bergaust L. Liu B. Frostegård Å (2012). Regulation of denitrification at the cellular level: A clue to the understanding of N_2_O emissions from soils. *Philos. Trans. R. Soc. B Biol. Sci.* 367 1226–1234. 10.1098/rstb.2011.0321 22451108 PMC3306626

[B3] Banerjee S. Zhao C. Garland G. Edlinger A. García-Palacios P. Romdhane S. (2024). Biotic homogenization, lower soil fungal diversity and fewer rare taxa in arable soils across Europe. *Nat. Commun.* 15:327. 10.1038/s41467-023-44073-6 38184663 PMC10771452

[B4] Barnard R. Leadley P. W. Hungate B. A. (2005). Global change, nitrification, and denitrification: A review. *Glob. Biogeochem. Cycles* 19:GB1007. 10.1029/2004GB002282

[B5] Bhattarai H. R. Wanek W. Siljanen H. M. P. Ronkainen J. G. Liimatainen M. Hu Y. (2021). Denitrification is the major nitrous acid production pathway in boreal agricultural soils. *Commun. Earth Environ.* 2:54. 10.1038/s43247-021-00125-7

[B6] Blackmer A. M. Bremner J. M. (1978). Inhibitory effect of nitrate on reduction of N_2_O to N_2_ by soil microorganisms. *Soil Biol. Biochem.* 10 187–191. 10.1016/0038-0717(78)90095-0

[B7] Bolyen E. Rideout J. R. Dillon M. R. Bokulich N. A. Abnet C. C. Al-Ghalith G. A. (2019). Reproducible, interactive, scalable and extensible microbiome data science using QIIME 2. *Nat. Biotechnol.* 37 852–857. 10.1038/s41587-019-0209-9 31341288 PMC7015180

[B8] Bösch Y. Pold G. Saghaï A. Karlsson M. Jones C. M. Hallin S. (2023). Distribution and environmental drivers of fungal denitrifiers in global soils. *Microbiol. Spectr.* 11:e00061-23. 10.1128/spectrum.00061-23 37222601 PMC10269876

[B9] Braker G. Schwarz J. Conrad R. (2010). Influence of temperature on the composition and activity of denitrifying soil communities. *FEMS Microbiol. Ecol.* 73 134–148. 10.1111/j.1574-6941.2010.00884.x 20455938

[B10] Callahan B. J. McMurdie P. J. Rosen M. J. Han A. W. Johnson A. J. Holmes S. P. (2016). DADA2: High-resolution sample inference from Illumina amplicon data. *Nat. Methods* 13 581–583. 10.1038/nmeth.3869 27214047 PMC4927377

[B11] Chen H. Mothapo N. V. Shi W. (2014). The significant contribution of fungi to soil N_2_O production across diverse ecosystems. *Appl. Soil Ecol.* 73 70–77. 10.1016/j.apsoil.2013.08.011

[B12] Choma C. Clavel T. Dominguez H. Razafindramboa N. Soumille H. Nguyen-the C. (2000). Effect of temperature on growth characteristics of *Bacillus cereus* TZ415. *Int. J. Food Microbiol.* 55 73–77. 10.1016/S0168-1605(00)00197-5 10791720

[B13] Chunyi K. Wei S. Mingken W. Chunyu X. Changxiu L. (2024). Diversity, community structure, and abundance of *nirS*-type denitrifying bacteria on suspended particulate matter in coastal high-altitude aquaculture pond water. *Sci. Rep.* 14:5594. 10.1038/s41598-024-56196-x 38454013 PMC10920899

[B14] Crenshaw C. L. Lauber C. Sinsabaugh R. L. Stavely L. K. (2008). Fungal control of nitrous oxide production in semiarid grassland. *Biogeochemistry* 87 17–27. 10.1007/s10533-007-9165-4

[B15] Čuhel J. Šimek M. (2011). Proximal and distal control by pH of denitrification rate in a pasture soil. *Agric. Ecosyst. Environ.* 141 230–233. 10.1016/j.agee.2011.02.016

[B16] Dai Z. Yu M. Chen H. Zhao H. Huang Y. Su W. (2020). Elevated temperature shifts soil N cycling from microbial immobilization to enhanced mineralization, nitrification and denitrification across global terrestrial ecosystems. *Glob. Change Biol.* 26 5267–5276. 10.1111/gcb.15211 32614503

[B17] Deng N. Gubry-Rangin C. Song X.-T. Ju X.-T. Liu S.-Y. Shen J.-P. (2024). AOB *Nitrosospira* cluster 3a.2 (D11) dominates N_2_O emissions in fertilised agricultural soils. *J. Environ. Manage.* 355:120504. 10.1016/j.jenvman.2024.120504 38447513

[B18] Firestone M. K. Firestone R. B. Tiedje J. M. (1980). Nitrous oxide from soil denitrification: Factors controlling its biological production. *Science* 208 749–751. 10.1126/science.208.4445.749 17771133

[B19] Hao J. Feng Y. Wang X. Yu Q. Zhang F. Yang G. (2022). Soil microbial nitrogen-cycling gene abundances in response to crop diversification: A meta-analysis. *Sci. Total Environ.* 838:156621. 10.1016/j.scitotenv.2022.156621 35691356

[B20] Hartmann M. Six J. (2023). Soil structure and microbiome functions in agroecosystems. *Nat. Rev. Earth Environ.* 4 4–18. 10.1038/s43017-022-00366-w

[B21] Herold M. B. Baggs E. M. Daniell T. J. (2012). Fungal and bacterial denitrification are differently affected by long-term pH amendment and cultivation of arable soil. *Soil Biol. Biochem.* 54 25–35. 10.1016/j.soilbio.2012.04.031

[B22] Higgins S. A. Welsh A. Orellana Luis H. Konstantinidis Konstantinos T. Chee-Sanford Joanne C. Sanford Robert A. (2016). Detection and diversity of fungal nitric oxide reductase genes (*p450nor*) in agricultural soils. *Appl. Environ. Microbiol.* 82 2919–2928. 10.1128/AEM.00243-16 26969694 PMC4959062

[B23] Hiis E. G. Vick S. H. W. Molstad L. Røsdal K. Jonassen K. R. Winiwarter W. (2024). Unlocking bacterial potential to reduce farmland N_2_O emissions. *Nature* 630 421–428. 10.1038/s41586-024-07464-3 38811724 PMC11168931

[B24] Huang Y. Xiao X. Long X. (2017). Fungal denitrification contributes significantly to N_2_O production in a highly acidic tea soil. *J. Soils Sediments* 17 1599–1606. 10.1007/s11368-017-1655-y

[B25] Kamp A. Høgslund S. Risgaard-Petersen N. Stief P. (2015). Nitrate storage and dissimilatory nitrate reduction by eukaryotic microbes. *Front. Microbiol.* 6:1492. 10.3389/fmicb.2015.01492 26734001 PMC4686598

[B26] Keuschnig C. Gorfer M. Li G. Mania D. Frostegard A. Bakken L. (2020). NO and N_2_O transformations of diverse fungi in hypoxia: Evidence for anaerobic respiration only in *Fusarium* strains. *Environ. Microbiol.* 22 2182–2195. 10.1111/1462-2920.14980 32157782

[B27] Kobayashi M. Matsuo Y. Takimoto A. Suzuki S. Maruo F. Shoun H. (1996). Denitrification, a novel type of respiratory metabolism in fungal mitochondrion*. *J. Biol. Chem.* 271 16263–16267. 10.1074/jbc.271.27.16263 8663075

[B28] Koskinen W. C. Keeney D. R. (1982). Effect of pH on the rate of gaseous products of denitrification in a silt loam soil. *Soil Sci. Soc. Am. J.* 46 1165–1167. 10.2136/sssaj1982.03615995004600060009x

[B29] Krichels A. H. Sanford R. A. Chee-Sanford J. C. Connor L. Van Allen R. Kent A. D. (2025). Distinct N-cycling microbial communities contribute to microtopographic variation in soil N_2_O emissions from denitrification. *Soil Biol. Biochem.* 202:109683. 10.1016/j.soilbio.2024.109683

[B30] Lauber C. L. Hamady M. Knight R. Fierer N. (2009). Pyrosequencing-based assessment of soil pH as a predictor of soil bacterial community structure at the continental scale. *Appl. Environ. Microbiol.* 75 5111–5120. 10.1128/AEM.00335-09 19502440 PMC2725504

[B31] Laughlin R. Stevens R. J. (2002). Evidence for fungal dominance of denitrification and codenitrification in a grassland soil. *Soil Sci. Soc. Am. J.* 66 1540–1548. 10.2136/sssaj2002.1540

[B32] Li H. Hong Y. W. Gao M. K. An X. L. Yang X. R. Zhu Y. G. (2023). Distinct responses of airborne abundant and rare microbial communities to atmospheric changes associated with Chinese New Year. *Imeta* 2:e140. 10.1002/imt2.140 38868217 PMC10989829

[B33] Li H. Yang X. Weng B. Su J. Nie S. Gilbert J. A. (2016). The phenological stage of rice growth determines anaerobic ammonium oxidation activity in rhizosphere soil. *Soil Biol. Biochem.* 100 59–65. 10.1016/j.soilbio.2016.05.015

[B34] Li H. Zhao S. Gao M.-K. Zhou Y. Xu B. Yang L.-Y. (2025). Experimental evidence for viral impact on microbial community, nitrification, and denitrification in an agriculture soil. *J. Hazard. Mater.* 489:137532. 10.1016/j.jhazmat.2025.137532 39933460

[B35] Li Y. Sun J. Tian D. Wang J. Ha D. Qu Y. (2018). Soil acid cations induced reduction in soil respiration under nitrogen enrichment and soil acidification. *Sci. Total Environ.* 615 1535–1546. 10.1016/j.scitotenv.2017.09.131 28927809

[B36] Long A. Heitman J. Tobias C. Philips R. Song B. (2013). Co-occurring anammox, denitrification, and codenitrification in agricultural soils. *Appl. Environ. Microbiol.* 79 168–176. 10.1128/AEM.02520-12 23087029 PMC3536082

[B37] Maeda K. Spor A. Edel-Hermann V. Heraud C. Breuil M.-C. Bizouard F. (2015). N_2_O production, a widespread trait in fungi. *Sci. Rep.* 5:9697. 10.1038/srep09697 25894103 PMC4403702

[B38] Margalef-Marti R. Thibault de Chanvalon A. Anschutz P. Amouroux D. Sebilo M. (2024). Synergies of chemodenitrification and denitrification in a saline inland lake. *Chemosphere* 359:142292. 10.1016/j.chemosphere.2024.142292 38729442

[B39] Molstad L. Dörsch P. Bakken L. R. (2007). Robotized incubation system for monitoring gases (O_2_, NO, N_2_O N_2_O) in denitrifying cultures. *J. Microbiol. Methods* 71 202–211. 10.1016/j.mimet.2007.08.011 17904668

[B40] Montzka S. A. Dlugokencky E. J. Butler J. H. (2011). Non-CO_2_ greenhouse gases and climate change. *Nature* 476 43–50. 10.1038/nature10322 21814274

[B41] Mothapo N. V. Chen H. Cubeta M. A. Shi W. (2013). Nitrous oxide producing activity of diverse fungi from distinct agroecosystems. *Soil Biol. Biochem.* 66 94–101. 10.1016/j.soilbio.2013.07.004

[B42] Mothapo N. Chen H. Cubeta M. A. Grossman J. M. Fuller F. Shi W. (2015). Phylogenetic, taxonomic and functional diversity of fungal denitrifiers and associated N_2_O production efficacy. *Soil Biol. Biochem.* 83 160–175. 10.1016/j.soilbio.2015.02.001

[B43] Olaya-Abril A. Hidalgo-Carrillo J. Luque-Almagro V. M. Fuentes-Almagro C. Urbano F. J. Moreno-Vivián C. (2021). Effect of pH on the denitrification proteome of the soil bacterium *Paracoccus denitrificans* PD1222. *Sci. Rep.* 11:17276. 10.1038/s41598-021-96559-2 34446760 PMC8390676

[B44] Pan Y. She D. Shi Z. Cao T. Xia Y. Shan J. (2023). Salinity and high pH reduce denitrification rates by inhibiting denitrifying gene abundance in a saline-alkali soil. *Sci. Rep.* 13:2155. 10.1038/s41598-023-29311-7 36750752 PMC9905596

[B45] Philippot L. Andert J. Jones C. M. Bru D. Hallin S. (2011). Importance of denitrifiers lacking the genes encoding the nitrous oxide reductase for N_2_O emissions from soil. *Global Change Biol.* 17 1497–1504. 10.1111/j.1365-2486.2010.02334.x

[B46] Pruesse E. Quast C. Knittel K. Fuchs B. M. Ludwig W. Peplies J. (2007). SILVA: A comprehensive online resource for quality checked and aligned ribosomal RNA sequence data compatible with ARB. *Nucleic Acids Res.* 35 7188–7196. 10.1093/nar/gkm864 17947321 PMC2175337

[B47] Qiu Y. Zhang Y. Zhang K. Xu X. Zhao Y. Bai T. (2024). Intermediate soil acidification induces highest nitrous oxide emissions. *Nat. Commun.* 15:2695. 10.1038/s41467-024-46931-3 38538640 PMC10973416

[B48] Ravishankara A. R. Daniel J. S. Portmann R. W. (2009). Nitrous Oxide (N_2_O): The dominant ozone-depleting substance emitted in the 21st century. *Science* 326 123–125. 10.1126/science.1176985 19713491

[B49] Risgaard-Petersen N. Langezaal A. Ingvardsen S. (2006). Evidence for complete denitrification in a benthic foraminifer. *Nature* 443 93–96. 10.1038/nature05070 16957731

[B50] Ryden J. C. (1981). N_2_O exchange between a grassland soil and the atmosphere. *Nature* 292 235–237. 10.1038/292235a0

[B51] Salmaso N. Boscaini A. Pindo M. (2020). Unraveling the diversity of eukaryotic microplankton in a large and deep perialpine lake using a high throughput sequencing approach. *Front. Microbiol.* 11:168. 10.3389/fmicb.2020.00789 32457713 PMC7221148

[B52] Schulz S. Kölbl A. Ebli M. Buegger F. Schloter M. Fiedler S. (2017). Field-scale pattern of denitrifying microorganisms and N_2_O emission rates indicate a high potential for complete denitrification in an agriculturally used organic soil. *Microb. Ecol.* 74 765–770. 10.1007/s00248-017-0991-1 28492990

[B53] Senbayram M. Budai A. Bol R. Chadwick D. Marton L. Gündogan R. (2019). Soil NO3^–^ level and O_2_ availability are key factors in controlling N_2_O reduction to N_2_ following long-term liming of an acidic sandy soil. *Soil Biol. Biochem.* 132 165–173. 10.1016/j.soilbio.2019.02.009

[B54] Sennett L. B. Roco C. A. Lim N. Y. N. Yavitt J. B. Dörsch P. Bakken L. R. (2024). Determining how oxygen legacy affects trajectories of soil denitrifier community dynamics and N_2_O emissions. *Nat. Commun.* 15:7298. 10.1038/s41467-024-51688-w 39181870 PMC11344836

[B55] Shao S. Li Y. Li Z. Ma X. Zhu Y. Luo Y. (2024). Impact of tea tree cultivation on soil microbiota, soil organic matter, and nitrogen cycling in mountainous plantations. *Agronomy* 14:638. 10.3390/agronomy14030638

[B56] Shoun H. Suyama W. Yasui T. (1989). Soluble, nitrate/nitrite-inducible cytochrome P-450 of the fungus, *Fusarium oxysporum*. *FEBS Lett.* 244 11–14. 10.1016/0014-5793(89)81151-2 2924900

[B57] ŠImek M. Cooper J. E. (2002). The influence of soil pH on denitrification: Progress towards the understanding of this interaction over the last 50 years. *Eur. J. Soil Sci.* 53 345–354. 10.1046/j.1365-2389.2002.00461.x

[B58] Su X. Li G. Cotner J. B. Wei L. Wang Y. Pan T. (2021). Long-term organic fertilization changes soil active bacterial composition and multifunctionality: RNA-based bacterial community and qPCR-based SmartChip analysis. *J. Soils Sediments* 21 799–809. 10.1007/s11368-020-02854-2

[B59] Tan Y. Chen Z. Liu W. Yang M. Du Z. Wang Y. (2024). Grazing exclusion alters denitrification N_2_O/(N_2_O + N_2_) ratio in alpine meadow of Qinghai–Tibet Plateau. *Sci. Total Environ.* 912:169358. 10.1016/j.scitotenv.2023.169358 38135064

[B60] Taylor A. E. Myrold D. D. Bottomley P. J. (2019). Temperature affects the kinetics of nitrite oxidation and nitrification coupling in four agricultural soils. *Soil Biol. Biochem.* 136:107523. 10.1016/j.soilbio.2019.107523

[B61] Wang C. Kuzyakov Y. (2024). Mechanisms and implications of bacterial–fungal competition for soil resources. *ISME J.* 18:wrae073. 10.1093/ismejo/wrae073 38691428 PMC11104273

[B62] Wang L. Zheng B. Nan B. Hu P. (2014). Diversity of bacterial community and detection of *nirS*- and *nirK*-encoding denitrifying bacteria in sandy intertidal sediments along Laizhou Bay of Bohai Sea, China. *Mar. Pollut. Bull.* 88 215–223. 10.1016/j.marpolbul.2014.09.002 25256298

[B63] Wang Q. Garrity G. M. Tiedje J. M. Cole J. R. (2007). Naive Bayesian classifier for rapid assignment of rRNA sequences into the new bacterial taxonomy. *Appl. Environ. Microbiol.* 73 5261–5267. 10.1128/aem.00062-07 17586664 PMC1950982

[B64] Xiong R. He X. Gao N. Li Q. Qiu Z. Hou Y. (2024). Soil pH amendment alters the abundance, diversity, and composition of microbial communities in two contrasting agricultural soils. *Microbiol. Spectr.* 12:e04165-23. 10.1128/spectrum.04165-23 38916324 PMC11302230

[B65] Xu H.-J. Wang X.-H. Li H. Yao H.-Y. Su J.-Q. Zhu Y.-G. (2014). Biochar impacts soil microbial community composition and nitrogen cycling in an acidic soil planted with rape. *Environ. Sci. Technol.* 48 9391–9399. 10.1021/es5021058 25054835

[B66] Xu H.-J. Yang X.-R. Li S. Xue X.-M. Chang S. Li H. (2019). Nitrogen inputs are more important than denitrifier abundances in controlling denitrification-derived N_2_O emission from both urban and agricultural soils. *Sci. Total Environ.* 650 2807–2817. 10.1016/j.scitotenv.2018.10.001 30373058

[B67] Xu X. Liu X. Li Y. Ran Y. Liu Y. Zhang Q. (2017). High temperatures inhibited the growth of soil bacteria and archaea but not that of fungi and altered nitrous oxide production mechanisms from different nitrogen sources in an acidic soil. *Soil Biol. Biochem.* 107 168–179. 10.1016/j.soilbio.2017.01.003

[B68] Yang H. Lyu W. Lu L. Shi X. Li N. Wang W. (2020). Biogeography of microbiome and short-chain fatty acids in the gastrointestinal tract of duck. *Poult. Sci.* 99 4016–4027. 10.1016/j.psj.2020.03.040 32731989 PMC7597935

[B69] Yang W. Zhang L. Yang Y. Xiang H. Yang P. (2024). Plant secondary metabolites-mediated plant defense against bacteria and fungi pathogens. *Plant Physiol. Biochem.* 217:109224. 10.1016/j.plaphy.2024.109224 39437667

[B70] Yao S. Ni J. Chen Q. Borthwick A. G. L. (2013). Enrichment and characterization of a bacteria consortium capable of heterotrophic nitrification and aerobic denitrification at low temperature. *Bioresour. Technol.* 127 151–157. 10.1016/j.biortech.2012.09.098 23131636

[B71] Zhang Q. Han P. Xu H. Wang Q. Xu G. (2022). Survival strategies of Nitrospira in a stable nitritation-denitritation system treating low-strength fermented wastewater. *Biochem. Eng. J.* 187:08674. 10.1016/j.bej.2022.108674

[B72] Zheng N. Yu Y. Wang J. Chapman S. J. Yao H. Zhang Y. (2020). The conversion of subtropical forest to tea plantation changes the fungal community and the contribution of fungi to N_2_O production. *Environ. Pollut.* 265:115106. 10.1016/j.envpol.2020.115106 32806403

