# Supplementary material for: Combined effects of acidification and warming on soil denitrification and microbial community
Source: Front Microbiol. 2025 Apr 2;16:1572497. doi: 10.3389/fmicb.2025.1572497 (PMC11999948; doi:10.3389/fmicb.2025.1572497)
Supplement: Supplementary file 1 [file Table_1.docx]

**Combined effects of acidification and warming on soil denitrification and microbial community**

**Pei-yuan Xu^1,2^, Mengke Gao^1,3^, Yu-chen Li^1, 2^, Jun Ye^2^, Jian-qiang Su^1,4^,** **Hu Li^1, 4*^**

^1^ State Key Laboratory for Ecological Security of Regions and Cities, Institute of Urban Environment, Chinese Academy of Sciences, Xiamen, China, 361021

^2^ School of Life Sciences, Hebei University, Baoding, China, 071002

^3^ College of JunCao Science and Ecology, Fujian Agriculture and Forestry University, Fuzhou, China, 350002

^4^ University of Chinese Academy of Sciences, Beijing, China, 100049

*** Correspondence:
Hu Li** E-mail: [hli@iue.ac.cn](mailto:hli@iue.ac.cn)

**Running Title: Impacts of acidification and warming on denitrification in soil**

**Manuscript length: 4121 words**

**Keywords:** Acidification, Warming, RNA level, Microbial community, Denitrification rate.

Supplementary Materials

## 1 Supplementary Figures


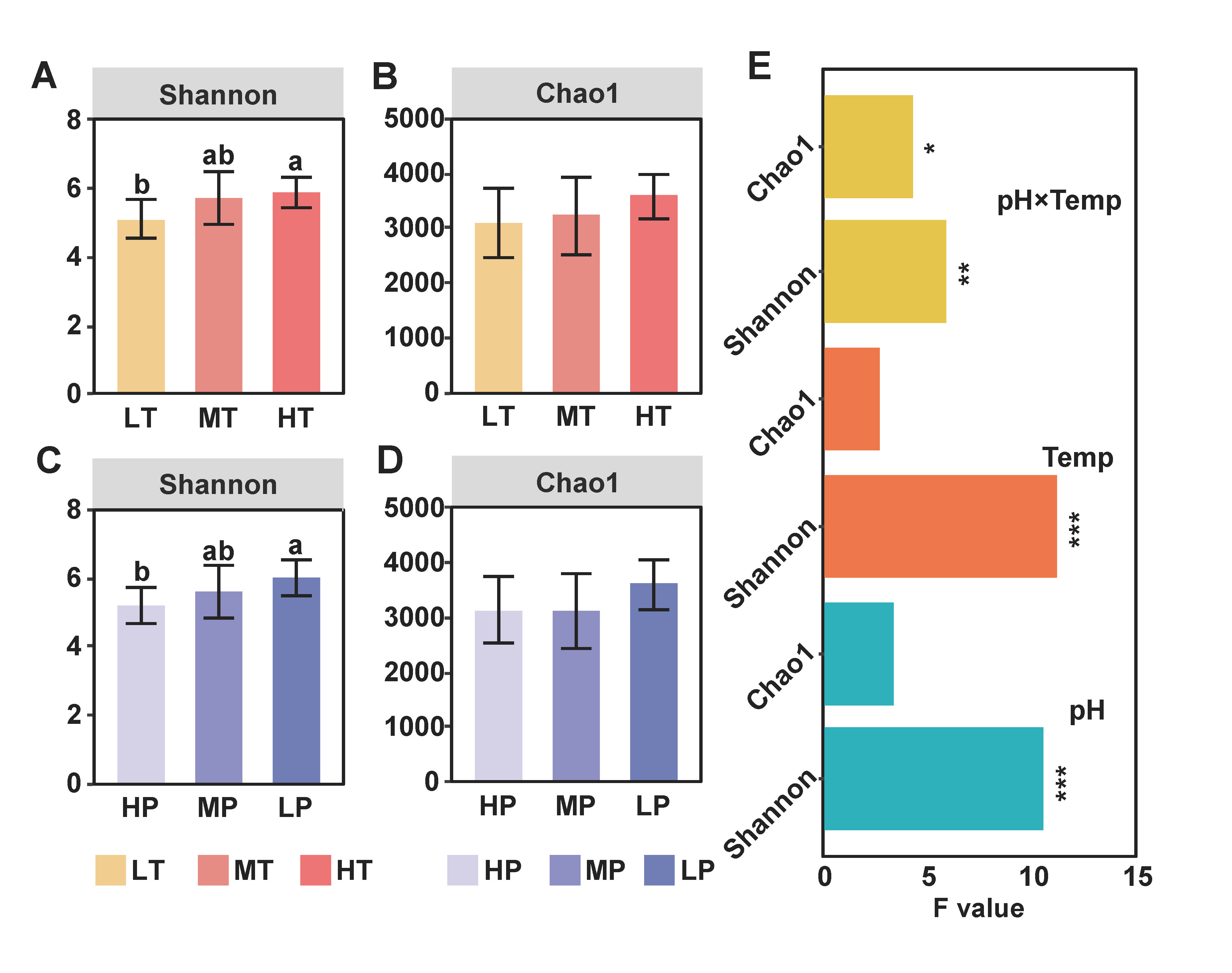


**Supplementary Figure 1.** The figures were based on an analysis of bacterial data. (A-D) Alpha diversity metrics for bacteria; these figures illustrated the variations in Shannon and Chao1 indices among treatment groups. (E) This figure presented the results of a two-factor ANOVA for bacteria.


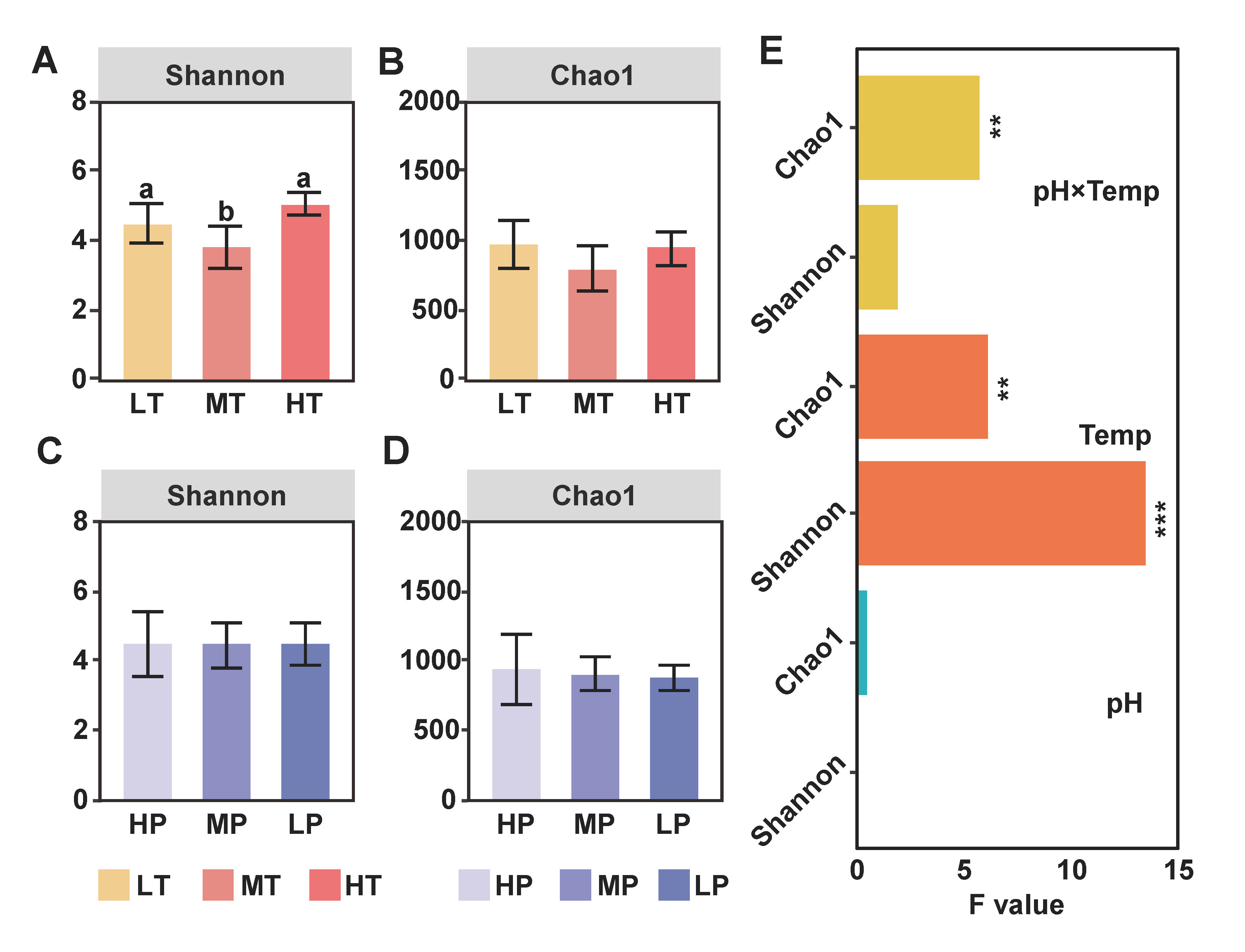


**Supplementary Figure 2.** The figures were based on an analysis of micro-eukaryotic data. (A-D) Alpha diversity metrics for micro-eukaryotes; these figures illustrated the variations in Shannon and Chao1 indices among treatment groups. (E) This figure presented the results of a two-factor ANOVA for eukaryotic microbes.


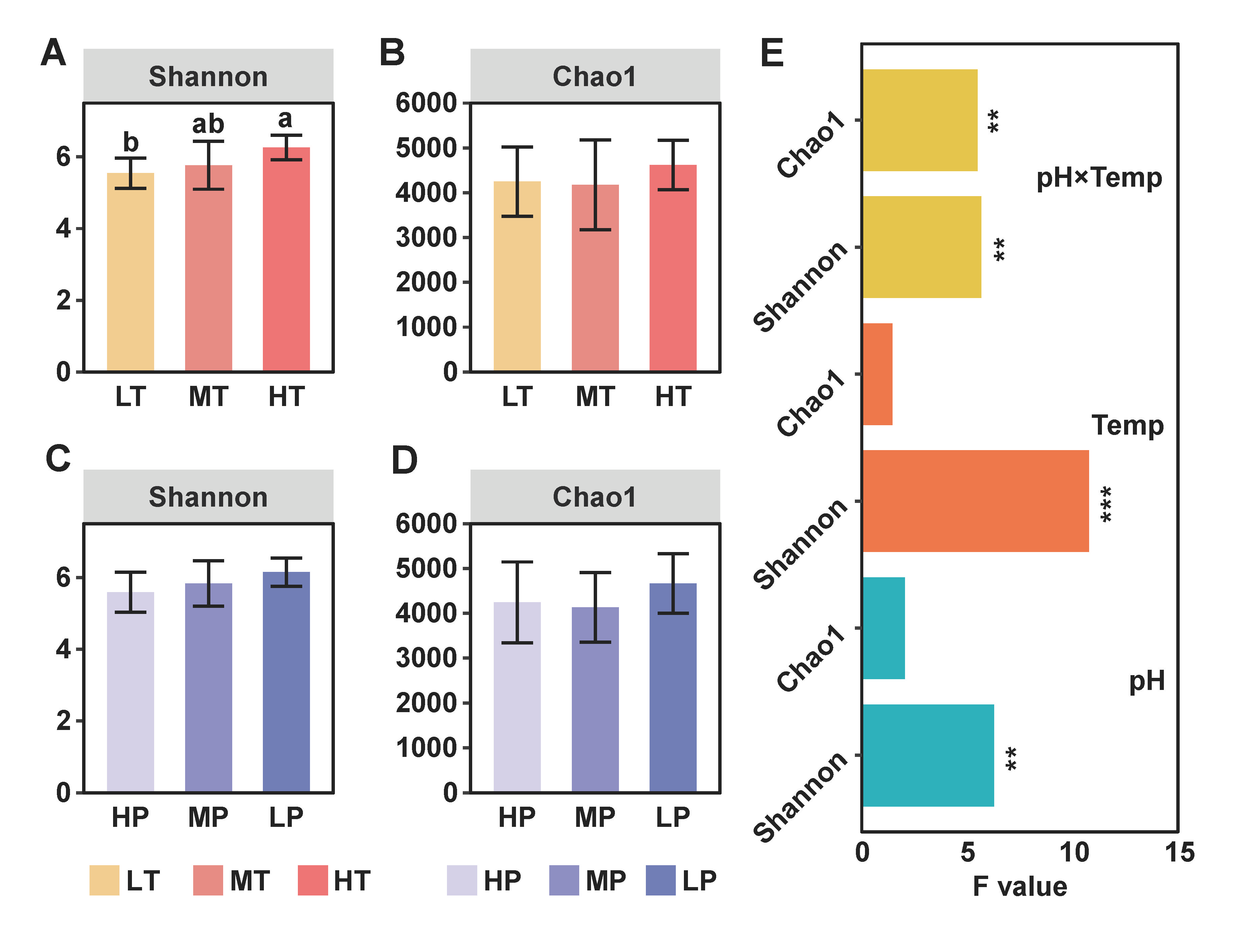


**Supplementary Figure 3.** The figures were based on an analysis of overall microbial data. (A-D) To analyze the overall diversity distribution of microbial communities in soil, all Amplicon Sequence Variants (ASVs) from 16S and 18S rRNA gene sequences were combined into a single table. The overall microbial diversity indices were calculated from this combined table using R. (E) A two-way ANOVA was performed using the ASV table derived from soil samples to examine the effects of temperature, pH, and their interaction on overall microbial alpha diversity indices.


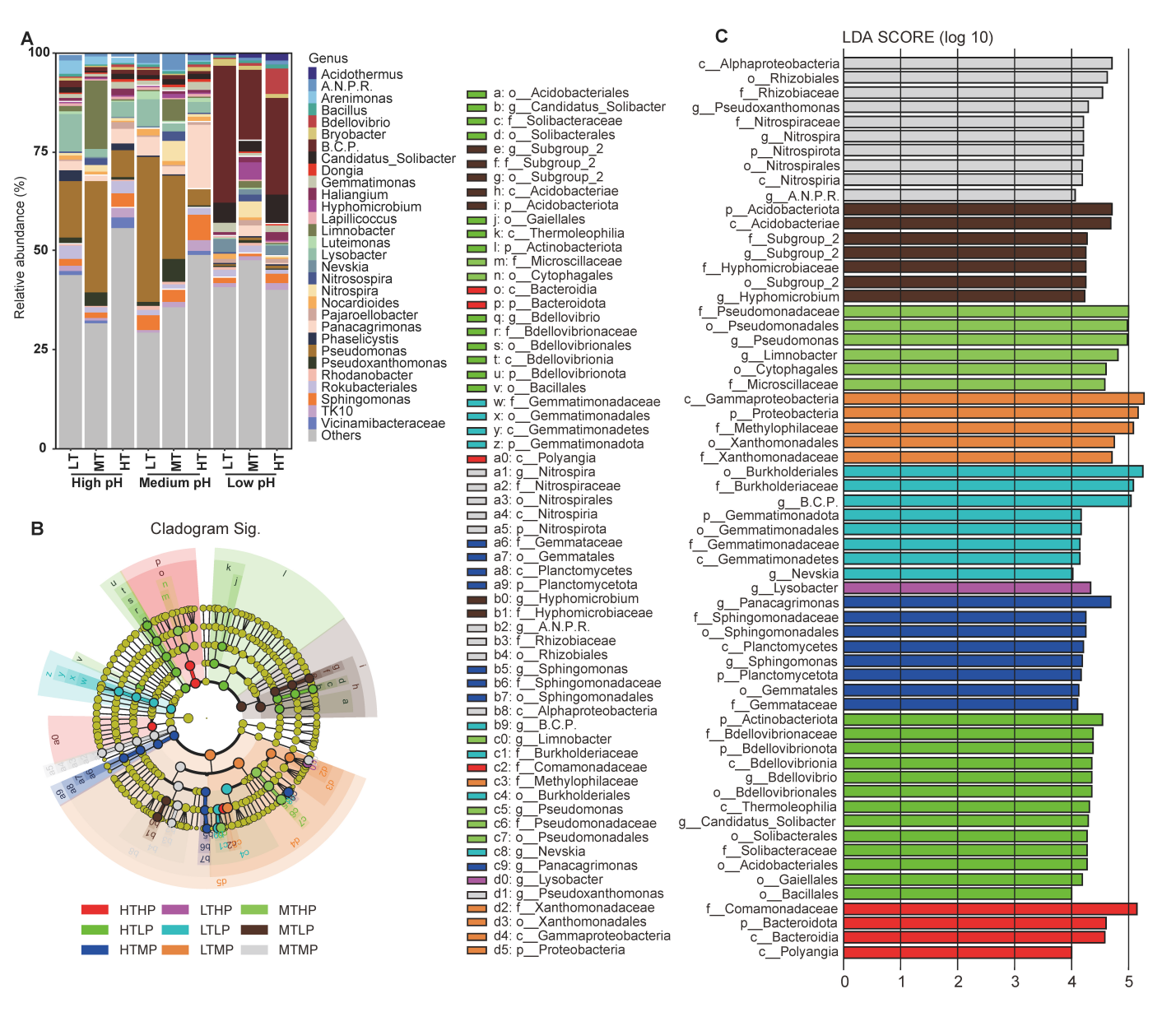


**Supplementary Figure 4.** The figures were based on an analysis of bacterial data. (A) Relative abundance of the top thirty most abundant taxa at the genus level of bacteria under nine treatments. (B,C) The LEfSe method was employed to identify species exhibiting significant differences among treatment groups across phylum to genus levels, with a linear discriminant analysis (LDA) threshold set at 4. In the figure, the abbreviation A.N.P.R. was *Allorhizobium_Neorhizobium_Pararhizobium_Rhizobium*, while B.C.P. represented *Burkholderia_Caballeronia_Paraburkholderia*.


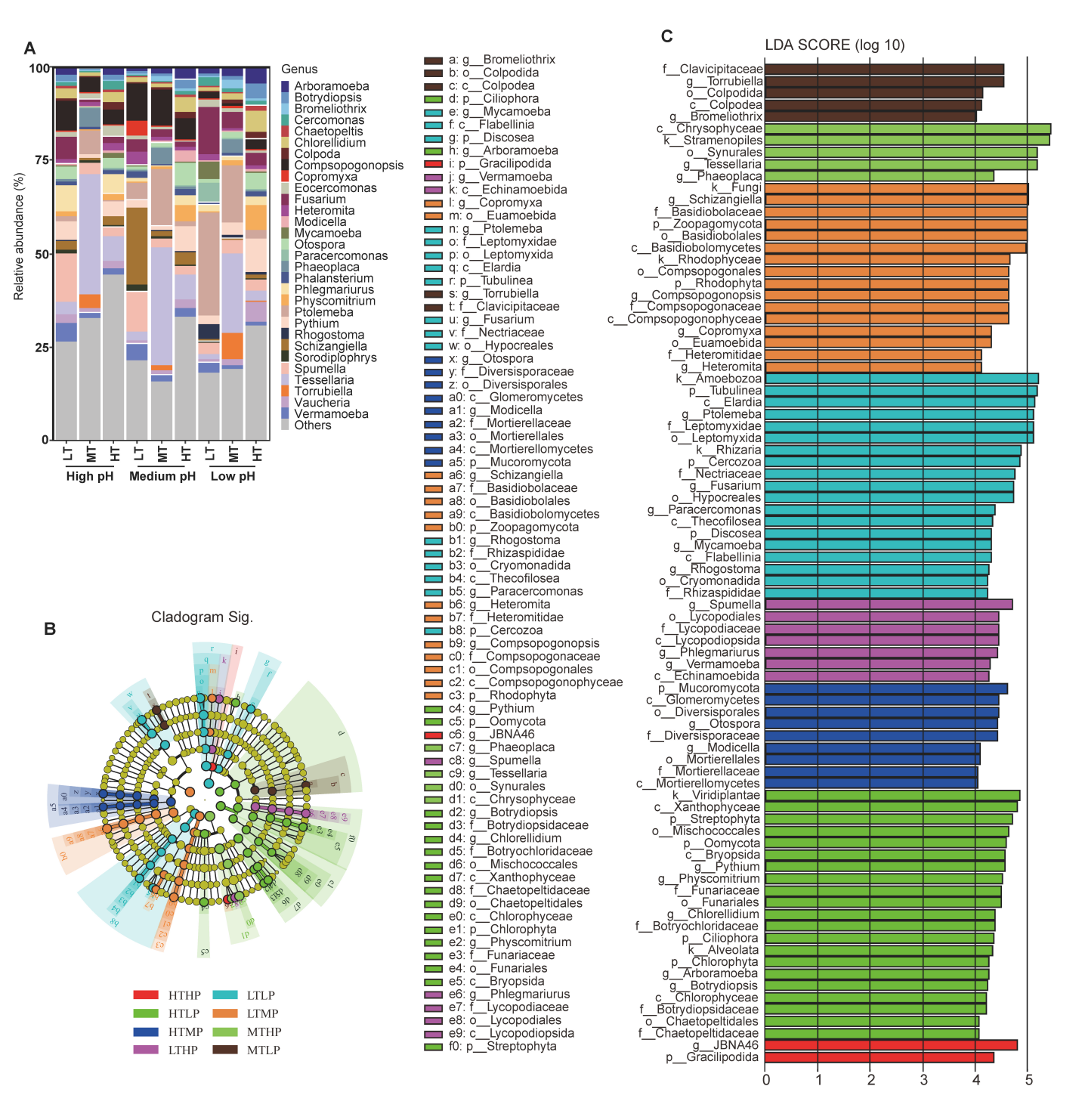


**Supplementary Figure 5.** The figures were based on an analysis of micro-eukaryotic data. (A) Relative abundance of the top thirty most abundant taxa at the genus level of eukaryotic microbes under nine treatments. (B,C) The LEfSe method identified species that exhibited significant differences among treatment groups across phylum to genus levels, with a threshold set at 4.


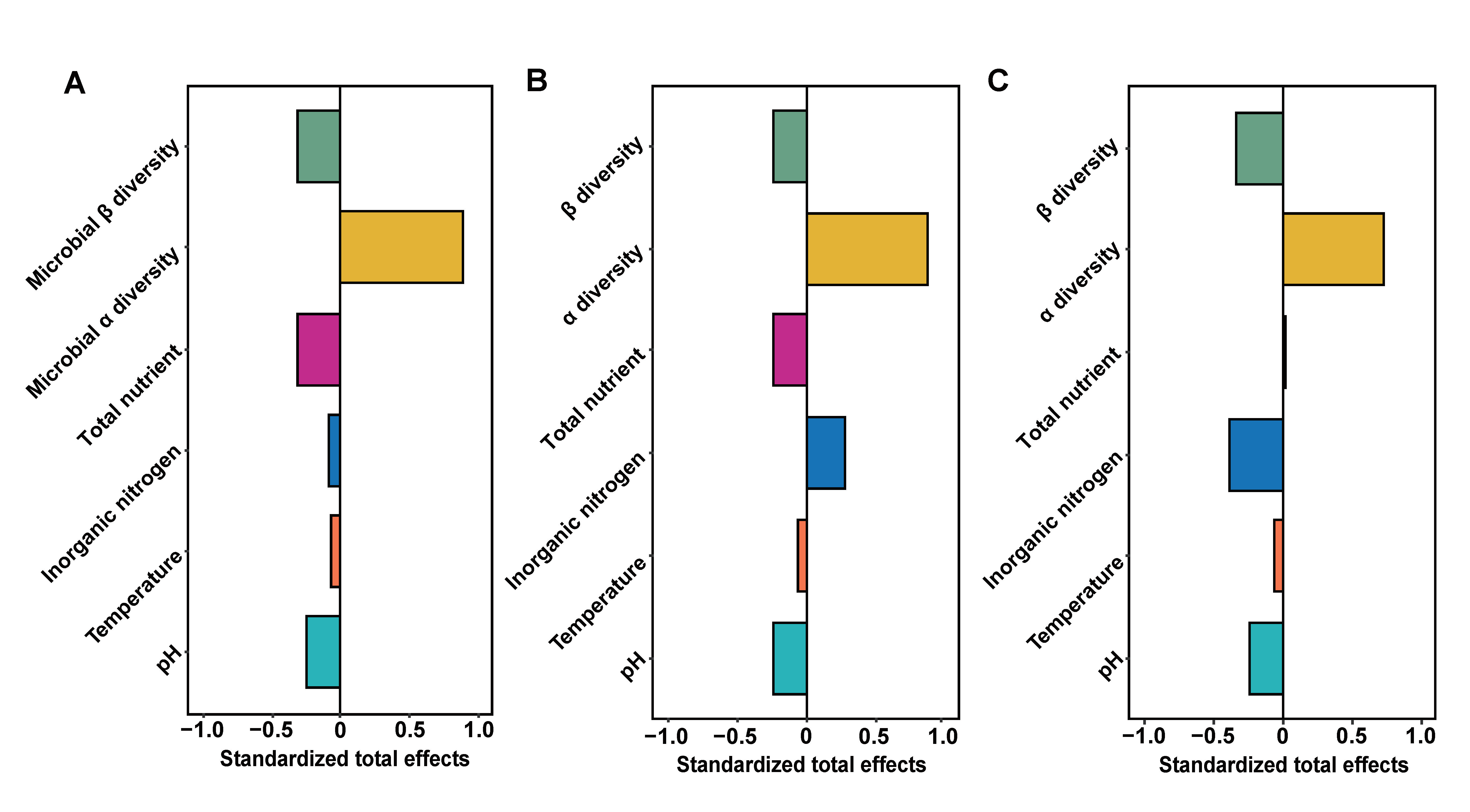


**Supplementary Figure 6.** (A-C) Standardized effects were obtained from the PLSPM model of overall, bacterial and eukaryotic microbial communities, respectively. We employed the Shannon and Chao1 indices to assess alpha diversity, while the PCoA1 axis was used to represent beta diversity.
